# (*E*)-2,2′-[3-(4-Fluoro­phen­yl)prop-2-ene-1,1-di­yl]bis­(3-hy­droxy-5,5-dimethyl­cyclo­hex-2-en-1-one)

**DOI:** 10.1107/S1600536813004364

**Published:** 2013-02-20

**Authors:** Joo Hwan Cha, Sun-Joon Min, Yong Seo Cho, Jae Kyun Lee, Junghwan Park

**Affiliations:** a Advanced Analysis Center, Korea Institute of Science & Technology, Hwarangro 14-gil, Seongbuk-gu, Seoul 136-791, Republic of Korea; b Center for Neuro-Medicine, Korea Institute of Science & Technology, Hwarangro 14-gil, Seongbuk-gu, Seoul 136-791, Republic of Korea; c Corporated R&D Center, Duksan Hi-Metal Co. Ltd, Cheonan-si, 331-821, Republic of Korea

## Abstract

In the title compound, C_25_H_29_FO_4_, each cyclo­hexenone ring has an envelope conformation with the dimethyl-substituted atom as the flap. The hy­droxy and carbonyl groups form two intra­molecular O—H⋯O hydrogen bonds, as is typical for xanthene derivatives. In the crystal, very weak C—H⋯O hydrogen bonds link mol­ecules into dimers.

## Related literature

For the crystal structures of related xanthenes derivatives, see: Cha *et al.* (2011 ▶, 2012 ▶).
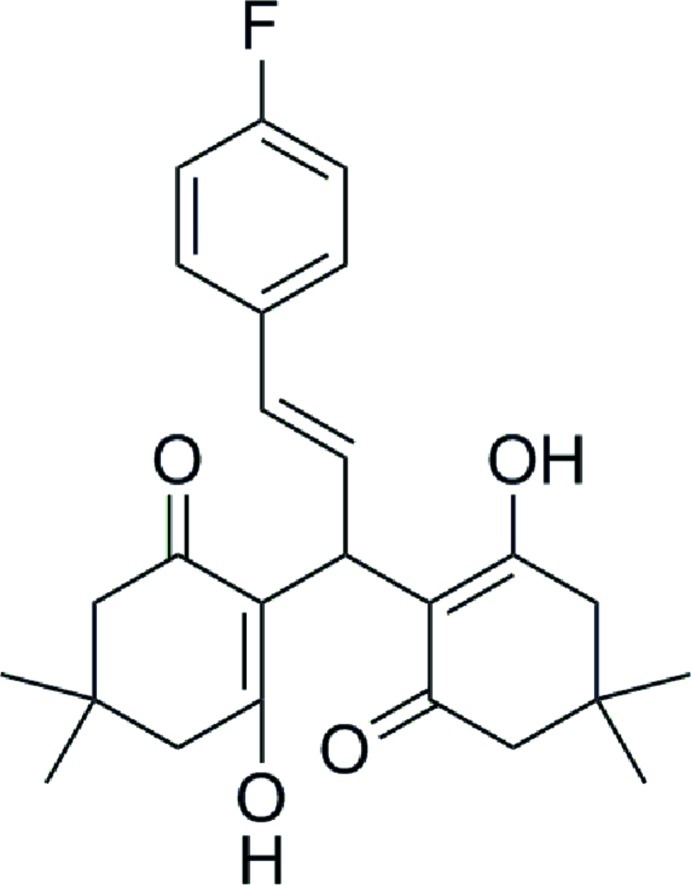


## Experimental

### 

#### Crystal data

C_25_H_29_FO_4_
*M* *_r_* = 412.50
Monoclinic, 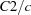

*a* = 26.1146 (13) Å
*b* = 9.6961 (4) Å
*c* = 20.5638 (9) Å
β = 121.5921 (15)°
*V* = 4435.3 (4) Å^3^
*Z* = 8
Mo *K*α radiation
μ = 0.09 mm^−1^
*T* = 296 K
0.30 × 0.20 × 0.10 mm

#### Data collection

Rigaku R-AXIS RAPID diffractometer
Absorption correction: multi-scan (*ABSCOR*; Rigaku, 1995 ▶) *T*_min_ = 0.771, *T*_max_ = 0.991
21193 measured reflections
5070 independent reflections
3370 reflections with *F* ^2^ > 2σ(*F* ^2^)
*R*_int_ = 0.026

#### Refinement

*R*[*F* ^2^ > 2σ(*F* ^2^)] = 0.048
*wR*(*F* ^2^) = 0.158
*S* = 1.09
5070 reflections
281 parameters
H-atom parameters constrained
Δρ_max_ = 0.38 e Å^−3^
Δρ_min_ = −0.26 e Å^−3^

### 

Data collection: *RAPID-AUTO* (Rigaku, 2006 ▶); cell refinement: *RAPID-AUTO*; data reduction: *RAPID-AUTO*; program(s) used to solve structure: *Il Milione* (Burla *et al.*, 2007 ▶); program(s) used to refine structure: *SHELXL97* (Sheldrick, 2008 ▶); molecular graphics: *CrystalStructure* (Rigaku, 2010 ▶); software used to prepare material for publication: *CrystalStructure*.

## Supplementary Material

Crystal structure: contains datablock(s) global, I. DOI: 10.1107/S1600536813004364/cv5387sup1.cif

Structure factors: contains datablock(s) I. DOI: 10.1107/S1600536813004364/cv5387Isup2.hkl

Supplementary material file. DOI: 10.1107/S1600536813004364/cv5387Isup3.cml

Additional supplementary materials:  crystallographic information; 3D view; checkCIF report

## Figures and Tables

**Table 1:** Hydrogen-bond geometry (Å, °)

*D*—H⋯*A*	*D*—H	H⋯*A*	*D*⋯*A*	*D*—H⋯*A*
O1—H1*A*⋯O4	0.82	1.78	2.582 (2)	168
O3—H3⋯O2	0.82	1.85	2.652 (3)	168
C9—H9⋯O1^i^	0.93	2.62	3.353 (3)	136
C16—H16⋯O4^i^	0.93	2.62	3.499 (3)	158

Symmetry code: (i) 
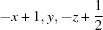
.

**Fig. 1.**
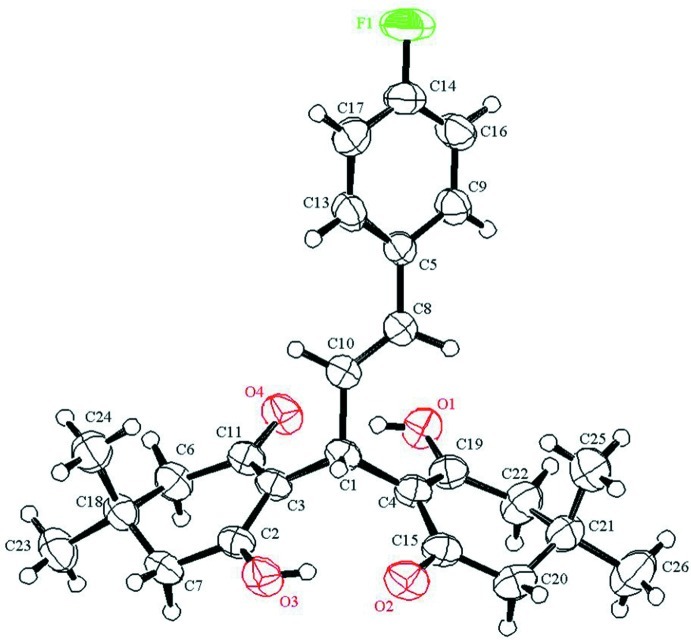
The molecular structure of (I) showing the atomic numbering and 50% probability displacement ellipsoid.

## supplementary crystallographic information

### Comment

As a part of our ongoing study of the substituent effect on the solide state structures of two cyclohexenone ring derivatives (Cha *et al.*, 2011, 2012), we present here the title compound (I).

In (I) (Fig. 1), the bond lengths and angles are normal and correspond to those observed in related structures (Cha *et al.*, 2011, 2012). Two cyclohexenone rings show an envelope conformation. The dihedral angle between the cyclohexenone mean planes is 41.08 (75)° while the dihedral angles between the benzene ring and the two cyclohexenone mean planes are 53.97 (75)° and 79.79 (14)°, respectively. In the crystal, weak intermolecular C—H···O hydrogen bonds (Table 1) link molecules into centrosymmetric dimers.

### Experimental

To solution of 5,5-Dimethyl-1,3-cyclohexanedione (4.61 mmol), 4-fluorocinnamaldehyde(1.84 mmol) and 4 Å MS was added catalytic amounts of *L*-proline in under nitrogen atmosphere. The anhydrous ethyl acetate (2 ml) was added to a reaction mixture and the solution was stirred at room temperature for 10 h. The progress of reaction was monitored by TLC. After completion of reaction, the reaction mixture was filtered through pad of celite to remove MS and evaporation of the solvent afforded a mixture. The mixture was purified by flash column chromatography to afford the title compound as a colorless solid in yield 82%. Recrystallization from ethanol gave crystals suitable for X-ray analysis.

### Refinement

All hydrogen atoms were positioned geometrically and refined using a riding model with C—H = 0.93–0.97 Å and *U*iso(H) = 1.2 or 1.5 *U*eq(C).

### Crystal data

**Table:**

C_25_H_29_FO_4_	*F*(000) = 1760.00
*M**_r_* = 412.50	*D*_x_ = 1.235 Mg m^−^^3^
Monoclinic, *C*2/*c*	Mo *K*α radiation, λ = 0.71075 Å
Hall symbol: -C 2yc	Cell parameters from 14685 reflections
*a* = 26.1146 (13) Å	θ = 3.2–27.5°
*b* = 9.6961 (4) Å	µ = 0.09 mm^−^^1^
*c* = 20.5638 (9) Å	*T* = 296 K
β = 121.5921 (15)°	Block, colourless
*V* = 4435.3 (4) Å^3^	0.30 × 0.20 × 0.10 mm
*Z* = 8	

### Data collection

**Table:**

Rigaku R-AXIS RAPID diffractometer	3370 reflections with *F*^2^ > 2σ(*F*^2^)
Detector resolution: 10.000 pixels mm^-1^	*R*_int_ = 0.026
ω scans	θ_max_ = 27.5°
Absorption correction: multi-scan (*ABSCOR*; Rigaku, 1995)	*h* = −33→33
*T*_min_ = 0.771, *T*_max_ = 0.991	*k* = −10→12
21193 measured reflections	*l* = −26→26
5070 independent reflections	

### Refinement

**Table:**

Refinement on *F*^2^	Secondary atom site location: difference Fourier map
*R*[*F*^2^ > 2σ(*F*^2^)] = 0.048	Hydrogen site location: inferred from neighbouring sites
*wR*(*F*^2^) = 0.158	H-atom parameters constrained
*S* = 1.09	*w* = 1/[σ^2^(*F*_o_^2^) + (0.0853*P*)^2^ + 0.9138*P*] where *P* = (*F*_o_^2^ + 2*F*_c_^2^)/3
5070 reflections	(Δ/σ)_max_ < 0.001
281 parameters	Δρ_max_ = 0.38 e Å^−^^3^
0 restraints	Δρ_min_ = −0.26 e Å^−^^3^
Primary atom site location: structure-invariant direct methods	

### Special details

**Table:**

Geometry. All e.s.d.'s (except the e.s.d. in the dihedral angle between two l.s. planes) are estimated using the full covariance matrix. The cell e.s.d.'s are taken into account individually in the estimation of e.s.d.'s in distances, angles and torsion angles; correlations between e.s.d.'s in cell parameters are only used when they are defined by crystal symmetry. An approximate (isotropic) treatment of cell e.s.d.'s is used for estimating e.s.d.'s involving l.s. planes.
Refinement. Refinement was performed using all reflections. The weighted *R*-factor (*wR*) and goodness of fit (*S*) are based on *F*^2^. *R*-factor (gt) are based on *F*. The threshold expression of *F*^2^ > 2.0 σ(*F*^2^) is used only for calculating *R*-factor (gt).

### Fractional atomic coordinates and isotropic or equivalent isotropic displacement parameters (Å^2^)

**Table:**

	*x*	*y*	*z*	*U*_iso_*/*U*_eq_	
F1	0.38293 (7)	0.14318 (15)	−0.00934 (8)	0.0871 (5)	
O1	0.46981 (5)	0.59709 (12)	0.36061 (8)	0.0510 (4)	
O2	0.28907 (6)	0.81844 (13)	0.29527 (8)	0.0559 (4)	
O3	0.26165 (6)	0.60434 (13)	0.35157 (8)	0.0541 (4)	
O4	0.43779 (6)	0.37861 (13)	0.40222 (8)	0.0528 (4)	
C1	0.33939 (7)	0.55677 (16)	0.29460 (9)	0.0374 (4)	
C2	0.30171 (7)	0.50860 (17)	0.38325 (9)	0.0410 (4)	
C3	0.34468 (7)	0.48284 (16)	0.36326 (9)	0.0374 (4)	
C4	0.37305 (7)	0.69315 (16)	0.31271 (9)	0.0394 (4)	
C5	0.37392 (7)	0.39250 (16)	0.14693 (9)	0.0397 (4)	
C6	0.39243 (8)	0.30729 (19)	0.47009 (10)	0.0478 (4)	
C7	0.30055 (9)	0.42833 (19)	0.44439 (10)	0.0490 (5)	
C8	0.37200 (8)	0.48601 (18)	0.20228 (10)	0.0435 (4)	
C9	0.41934 (8)	0.40838 (19)	0.13113 (10)	0.0468 (4)	
C10	0.34759 (7)	0.45888 (17)	0.24304 (9)	0.0401 (4)	
C11	0.39165 (7)	0.39240 (17)	0.40863 (9)	0.0412 (4)	
C13	0.33133 (8)	0.28989 (19)	0.10782 (10)	0.0492 (5)	
C14	0.37988 (9)	0.22567 (19)	0.04230 (11)	0.0533 (5)	
C15	0.34225 (8)	0.81682 (18)	0.30644 (10)	0.0449 (4)	
C16	0.42251 (9)	0.3253 (2)	0.07889 (11)	0.0541 (5)	
C17	0.33405 (10)	0.2057 (2)	0.05524 (11)	0.0567 (5)	
C18	0.33098 (8)	0.28697 (18)	0.46091 (10)	0.0467 (4)	
C19	0.43444 (8)	0.70204 (17)	0.34034 (10)	0.0446 (4)	
C20	0.37073 (9)	0.95534 (18)	0.31481 (12)	0.0566 (5)	
C21	0.42333 (9)	0.95511 (18)	0.30305 (12)	0.0533 (5)	
C22	0.46479 (9)	0.83836 (19)	0.34999 (13)	0.0596 (5)	
C23	0.34063 (11)	0.2288 (3)	0.53606 (12)	0.0654 (6)	
C24	0.29292 (10)	0.1860 (2)	0.39620 (12)	0.0598 (5)	
C25	0.39917 (12)	0.9295 (3)	0.21754 (13)	0.0734 (7)	
C26	0.45606 (12)	1.0926 (2)	0.32438 (16)	0.0802 (7)	
H1	0.2969	0.5834	0.2640	0.0448*	
H1A	0.4545	0.5316	0.3695	0.0612*	
H3	0.2730	0.6624	0.3329	0.0649*	
H6A	0.4190	0.3511	0.5190	0.0574*	
H6B	0.4092	0.2174	0.4711	0.0574*	
H7A	0.2590	0.4154	0.4298	0.0588*	
H7B	0.3202	0.4822	0.4910	0.0588*	
H8	0.3906 (8)	0.5785 (19)	0.2057 (10)	0.046 (5)*	
H9	0.4482	0.4766	0.1564	0.0562*	
H10	0.3290 (9)	0.3665 (19)	0.2366 (11)	0.049 (5)*	
H13	0.3004	0.2776	0.1171	0.0590*	
H16	0.4531	0.3370	0.0688	0.0649*	
H17	0.3054	0.1373	0.0294	0.0681*	
H20A	0.3844	0.9906	0.3655	0.0679*	
H20B	0.3404	1.0183	0.2783	0.0679*	
H22A	0.4950	0.8273	0.3365	0.0716*	
H22B	0.4854	0.8641	0.4035	0.0716*	
H23A	0.3030	0.2275	0.5339	0.0785*	
H23B	0.3687	0.2860	0.5778	0.0785*	
H23C	0.3562	0.1367	0.5435	0.0785*	
H24A	0.2882	0.2202	0.3495	0.0717*	
H24B	0.2541	0.1764	0.3904	0.0717*	
H24C	0.3125	0.0978	0.4080	0.0717*	
H25A	0.4321	0.9278	0.2093	0.0880*	
H25B	0.3719	1.0021	0.1877	0.0880*	
H25C	0.3785	0.8426	0.2025	0.0880*	
H26A	0.4855	1.0929	0.3098	0.0962*	
H26B	0.4757	1.1062	0.3785	0.0962*	
H26C	0.4277	1.1657	0.2983	0.0962*	

### Atomic displacement parameters (Å^2^)

**Table:**

	*U*^11^	*U*^22^	*U*^33^	*U*^12^	*U*^13^	*U*^23^
F1	0.1116 (12)	0.0874 (9)	0.0818 (10)	−0.0026 (8)	0.0642 (10)	−0.0334 (8)
O1	0.0345 (7)	0.0479 (7)	0.0673 (9)	0.0074 (5)	0.0243 (6)	0.0042 (6)
O2	0.0429 (8)	0.0621 (8)	0.0646 (9)	0.0186 (6)	0.0295 (7)	0.0060 (7)
O3	0.0441 (8)	0.0627 (8)	0.0656 (9)	0.0137 (6)	0.0357 (7)	0.0085 (7)
O4	0.0414 (7)	0.0620 (8)	0.0626 (8)	0.0166 (6)	0.0325 (7)	0.0123 (7)
C1	0.0297 (8)	0.0471 (9)	0.0356 (8)	0.0065 (7)	0.0173 (7)	0.0019 (7)
C2	0.0348 (9)	0.0497 (9)	0.0396 (9)	0.0011 (7)	0.0203 (8)	−0.0048 (7)
C3	0.0312 (8)	0.0470 (9)	0.0341 (8)	0.0038 (7)	0.0171 (7)	−0.0012 (7)
C4	0.0359 (9)	0.0440 (9)	0.0370 (8)	0.0066 (7)	0.0183 (7)	0.0001 (7)
C5	0.0425 (9)	0.0425 (9)	0.0376 (8)	0.0010 (7)	0.0233 (8)	0.0030 (7)
C6	0.0443 (10)	0.0582 (11)	0.0366 (9)	0.0051 (8)	0.0182 (8)	0.0064 (8)
C7	0.0500 (11)	0.0617 (11)	0.0465 (10)	−0.0002 (9)	0.0330 (9)	−0.0037 (9)
C8	0.0456 (10)	0.0447 (9)	0.0456 (9)	−0.0018 (8)	0.0276 (8)	−0.0010 (8)
C9	0.0423 (10)	0.0523 (10)	0.0505 (10)	−0.0049 (8)	0.0276 (9)	−0.0040 (8)
C10	0.0388 (9)	0.0448 (9)	0.0378 (9)	0.0026 (7)	0.0208 (8)	0.0008 (7)
C11	0.0345 (9)	0.0502 (9)	0.0381 (9)	0.0016 (7)	0.0184 (7)	−0.0021 (7)
C13	0.0482 (11)	0.0565 (10)	0.0516 (10)	−0.0086 (8)	0.0322 (9)	−0.0017 (8)
C14	0.0636 (12)	0.0534 (10)	0.0486 (10)	0.0056 (9)	0.0334 (10)	−0.0074 (9)
C15	0.0422 (10)	0.0504 (10)	0.0409 (9)	0.0109 (8)	0.0208 (8)	0.0018 (8)
C16	0.0505 (11)	0.0637 (12)	0.0612 (12)	0.0023 (9)	0.0383 (10)	−0.0039 (10)
C17	0.0617 (13)	0.0527 (11)	0.0549 (11)	−0.0141 (9)	0.0301 (10)	−0.0109 (9)
C18	0.0495 (11)	0.0544 (10)	0.0400 (9)	−0.0027 (8)	0.0260 (9)	0.0006 (8)
C19	0.0395 (9)	0.0458 (9)	0.0466 (10)	0.0051 (7)	0.0213 (8)	−0.0018 (8)
C20	0.0600 (13)	0.0452 (10)	0.0633 (12)	0.0092 (9)	0.0314 (11)	−0.0039 (9)
C21	0.0591 (12)	0.0418 (9)	0.0634 (12)	0.0028 (8)	0.0350 (10)	−0.0007 (9)
C22	0.0457 (11)	0.0515 (11)	0.0734 (14)	−0.0025 (9)	0.0254 (11)	−0.0042 (10)
C23	0.0763 (15)	0.0750 (14)	0.0540 (12)	−0.0025 (11)	0.0405 (12)	0.0084 (10)
C24	0.0588 (13)	0.0620 (12)	0.0555 (12)	−0.0088 (10)	0.0279 (10)	−0.0063 (10)
C25	0.0962 (19)	0.0677 (13)	0.0680 (14)	−0.0037 (13)	0.0512 (14)	0.0025 (11)
C26	0.0828 (18)	0.0496 (12)	0.106 (2)	−0.0079 (11)	0.0482 (16)	−0.0115 (13)

### Geometric parameters (Å, º)

**Table:**

F1—C14	1.365 (3)	C21—C26	1.519 (3)
O1—C19	1.288 (2)	O1—H1A	0.820
O2—C15	1.284 (3)	O3—H3	0.820
O3—C2	1.291 (2)	C1—H1	0.980
O4—C11	1.286 (3)	C6—H6A	0.970
C1—C3	1.524 (3)	C6—H6B	0.970
C1—C4	1.522 (3)	C7—H7A	0.970
C1—C10	1.519 (3)	C7—H7B	0.970
C2—C3	1.405 (4)	C8—H8	1.01 (2)
C2—C7	1.492 (3)	C9—H9	0.930
C3—C11	1.395 (2)	C10—H10	0.993 (19)
C4—C15	1.412 (3)	C13—H13	0.930
C4—C19	1.395 (3)	C16—H16	0.930
C5—C8	1.476 (3)	C17—H17	0.930
C5—C9	1.393 (4)	C20—H20A	0.970
C5—C13	1.392 (3)	C20—H20B	0.970
C6—C11	1.501 (3)	C22—H22A	0.970
C6—C18	1.527 (4)	C22—H22B	0.970
C7—C18	1.531 (3)	C23—H23A	0.960
C8—C10	1.318 (4)	C23—H23B	0.960
C9—C16	1.379 (4)	C23—H23C	0.960
C13—C17	1.386 (4)	C24—H24A	0.960
C14—C16	1.364 (3)	C24—H24B	0.960
C14—C17	1.369 (4)	C24—H24C	0.960
C15—C20	1.501 (3)	C25—H25A	0.960
C18—C23	1.537 (4)	C25—H25B	0.960
C18—C24	1.527 (3)	C25—H25C	0.960
C19—C22	1.500 (3)	C26—H26A	0.960
C20—C21	1.514 (4)	C26—H26B	0.960
C21—C22	1.513 (3)	C26—H26C	0.960
C21—C25	1.546 (4)		
			
O1···O4	2.582 (2)	H25A···H26C	2.9839
O1···C1	2.958 (2)	H25B···H26A	2.8412
O1···C3	3.478 (3)	H25B···H26B	3.5526
O1···C8	3.094 (2)	H25B···H26C	2.5181
O1···C10	3.1214 (18)	H25C···H26A	3.4790
O1···C11	3.345 (3)	H25C···H26C	3.5627
O2···O3	2.652 (3)	F1···H3^i^	3.5443
O2···C1	2.861 (3)	F1···H20A^i^	2.8996
O2···C2	3.432 (3)	F1···H23C^xi^	3.1345
O2···C3	3.539 (2)	F1···H24C^xi^	2.9076
O2···C19	3.586 (3)	F1···H26B^viii^	3.2540
O3···C1	2.863 (3)	O1···H6A^ii^	2.6989
O3···C4	3.504 (3)	O1···H9^iii^	2.6183
O3···C11	3.599 (3)	O1···H16^iii^	3.0762
O3···C15	3.403 (3)	O2···H1^v^	3.2037
O4···C1	2.9201 (19)	O2···H10^v^	2.84 (3)
O4···C2	3.594 (3)	O2···H24A^v^	2.7478
O4···C4	3.505 (2)	O2···H24C^ix^	3.4056
O4···C10	2.9649 (19)	O3···H10^v^	3.295 (18)
O4···C19	3.368 (3)	O3···H13^v^	2.6374
C2···C4	3.407 (3)	O3···H20B^vii^	2.7334
C2···C6	2.860 (3)	O3···H25B^vii^	3.2933
C2···C24	3.158 (3)	O4···H16^iii^	2.6202
C3···C15	3.432 (3)	O4···H26B^vi^	2.9496
C3···C18	2.919 (3)	O4···H26C^vi^	2.8821
C3···C19	3.375 (3)	C1···H24A^v^	3.4711
C3···C24	3.396 (3)	C1···H24B^v^	3.4705
C4···C8	3.021 (3)	C2···H20B^vii^	3.4491
C4···C11	3.410 (3)	C5···H7B^i^	3.0013
C4···C21	2.913 (3)	C5···H23B^i^	3.3995
C4···C25	3.308 (4)	C5···H26C^vi^	3.4528
C5···C14	2.761 (3)	C6···H22B^ii^	3.3179
C7···C11	2.856 (4)	C7···H23A^xii^	3.3259
C8···C19	3.203 (3)	C8···H23B^i^	3.3493
C9···C17	2.760 (3)	C8···H24B^v^	3.3632
C10···C11	3.038 (3)	C8···H26C^vi^	3.5574
C10···C13	3.059 (3)	C9···H1A^iii^	3.5108
C10···C19	3.155 (3)	C9···H6A^i^	3.2768
C11···C24	3.167 (4)	C9···H7B^i^	2.8835
C13···C16	2.761 (4)	C9···H23B^i^	3.1961
C15···C22	2.843 (4)	C10···H24B^v^	3.3756
C15···C25	3.097 (5)	C10···H26C^vi^	3.3555
C19···C20	2.857 (3)	C13···H7B^i^	3.1625
C19···C25	3.107 (3)	C13···H17^xiii^	3.2645
F1···O2^i^	3.4600 (19)	C13···H25B^vi^	3.1280
F1···C15^i^	3.378 (3)	C13···H26C^vi^	3.5780
F1···C20^i^	3.589 (4)	C14···H7B^i^	3.1374
O1···C6^ii^	3.5777 (18)	C14···H23C^xi^	3.5700
O1···C9^iii^	3.353 (3)	C14···H26A^viii^	3.4741
O1···C16^iii^	3.569 (3)	C14···H26B^viii^	3.4324
O2···F1^iv^	3.4600 (19)	C15···H24A^v^	3.3645
O2···C10^v^	3.502 (3)	C16···H1A^iii^	3.4456
O2···C24^v^	3.592 (3)	C16···H6A^i^	3.3549
O3···C13^v^	3.458 (3)	C16···H7B^i^	2.9717
O4···C16^iii^	3.499 (3)	C16···H26A^viii^	3.2142
O4···C26^vi^	3.358 (4)	C16···H26B^viii^	3.1474
C6···O1^ii^	3.5777 (18)	C17···H7B^i^	3.2451
C9···O1^iii^	3.353 (3)	C17···H13^xiii^	3.4469
C10···O2^vii^	3.502 (3)	C17···H17^xiii^	3.4602
C13···O3^vii^	3.458 (3)	C17···H23C^xi^	3.4005
C15···F1^iv^	3.378 (3)	C17···H25B^vi^	3.0778
C16···O1^iii^	3.569 (3)	C18···H23A^xii^	3.5590
C16···O4^iii^	3.499 (3)	C19···H6A^ii^	3.4183
C16···C26^viii^	3.535 (3)	C20···H24C^ix^	3.3082
C20···F1^iv^	3.589 (4)	C22···H6A^ii^	3.3583
C23···C25^iv^	3.556 (4)	C22···H6B^ii^	3.4608
C24···O2^vii^	3.592 (3)	C22···H25A^iii^	3.5958
C25···C23^i^	3.556 (4)	C23···H7A^xii^	3.3374
C26···O4^ix^	3.358 (4)	C23···H8^iv^	3.55 (2)
C26···C16^x^	3.535 (3)	C23···H23A^xii^	3.2657
F1···H16	2.5265	C23···H25A^iv^	3.4340
F1···H17	2.5341	C23···H25B^iv^	3.5689
O1···H8	2.749 (17)	C23···H25C^iv^	3.1025
O1···H22A	2.4503	C24···H1^vii^	3.0411
O1···H22B	2.6959	C24···H20A^vi^	3.3570
O2···H1	2.4058	C24···H23A^xii^	3.5806
O2···H3	1.8447	C25···H23A^i^	3.5987
O2···H20A	2.7014	C25···H23B^i^	3.2955
O2···H20B	2.4821	C25···H23C^i^	3.2059
O3···H1	2.4234	C26···H16^x^	3.2570
O3···H7A	2.4622	H1···O2^vii^	3.2037
O3···H7B	2.7150	H1···C24^v^	3.0411
O4···H1A	1.7755	H1···H20B^vii^	3.2784
O4···H6A	2.7041	H1···H24A^v^	2.5911
O4···H6B	2.4732	H1···H24B^v^	2.8692
O4···H10	3.090 (16)	H1···H24C^v^	3.1795
C1···H1A	2.5724	H1A···C9^iii^	3.5108
C1···H3	2.4700	H1A···C16^iii^	3.4456
C1···H8	2.78 (3)	H1A···H6A^ii^	3.0874
C2···H1	2.4971	H1A···H9^iii^	2.9050
C2···H6A	3.2475	H1A···H16^iii^	2.7913
C2···H24A	2.8580	H3···F1^iv^	3.5443
C2···H24B	3.4831	H3···H10^v^	3.0122
C3···H1A	2.8426	H3···H13^v^	2.8370
C3···H3	2.3869	H3···H20B^vii^	2.9758
C3···H6A	3.0234	H3···H24A^v^	3.2648
C3···H6B	3.2346	H6A···O1^ii^	2.6989
C3···H7A	3.2415	H6A···C9^iv^	3.2768
C3···H7B	3.0127	H6A···C16^iv^	3.3549
C3···H10	2.67 (3)	H6A···C19^ii^	3.4183
C3···H24A	2.8821	H6A···C22^ii^	3.3583
C4···H1A	2.3956	H6A···H1A^ii^	3.0874
C4···H3	2.8668	H6A···H9^iv^	3.0169
C4···H8	2.71 (3)	H6A···H16^iv^	3.1682
C4···H10	3.452 (18)	H6A···H22A^ii^	3.1425
C4···H20A	3.0406	H6A···H22B^ii^	2.9929
C4···H20B	3.2454	H6B···C22^ii^	3.4608
C4···H22A	3.2316	H6B···H20A^vi^	2.9153
C4···H22B	3.0167	H6B···H22A^ii^	3.4254
C4···H25C	2.7559	H6B···H22B^ii^	2.7239
C5···H10	2.66 (3)	H6B···H26B^vi^	3.3576
C5···H16	3.2585	H7A···C23^xii^	3.3374
C5···H17	3.2614	H7A···H17^v^	3.1063
C6···H7A	3.3010	H7A···H23A^xii^	2.5240
C6···H7B	2.7340	H7A···H23C^xii^	3.3690
C6···H23A	3.3140	H7A···H25B^vii^	3.0946
C6···H23B	2.6019	H7A···H25C^vii^	3.2487
C6···H23C	2.7206	H7B···C5^iv^	3.0013
C6···H24A	2.6832	H7B···C9^iv^	2.8835
C6···H24B	3.3376	H7B···C13^iv^	3.1625
C6···H24C	2.7056	H7B···C14^iv^	3.1374
C7···H3	3.0328	H7B···C16^iv^	2.9717
C7···H6A	2.7402	H7B···C17^iv^	3.2451
C7···H6B	3.3008	H7B···H9^iv^	3.3151
C7···H23A	2.6568	H7B···H16^iv^	3.4437
C7···H23B	2.7355	H7B···H17^v^	3.4281
C7···H23C	3.3399	H8···C23^i^	3.55 (2)
C7···H24A	2.7057	H8···H23A^i^	3.5752
C7···H24B	2.6945	H8···H23B^i^	2.7186
C7···H24C	3.3429	H8···H24B^v^	3.3563
C8···H1	2.9917	H9···O1^iii^	2.6183
C8···H1A	2.9829	H9···H1A^iii^	2.9050
C8···H9	2.6130	H9···H6A^i^	3.0169
C8···H13	2.6860	H9···H7B^i^	3.3151
C8···H25C	3.4618	H9···H9^iii^	3.3508
C9···H8	2.62 (3)	H9···H23B^i^	2.9511
C9···H13	3.2248	H10···O2^vii^	2.84 (3)
C10···H1A	2.7289	H10···O3^vii^	3.295 (18)
C10···H13	2.8230	H10···H3^vii^	3.0122
C11···H1	3.2688	H10···H20B^vi^	3.4585
C11···H1A	2.5590	H10···H26C^vi^	2.9353
C11···H7B	3.2294	H13···O3^vii^	2.6374
C11···H10	3.03 (2)	H13···C17^xiii^	3.4469
C11···H24A	2.8507	H13···H3^vii^	2.8370
C11···H24C	3.5218	H13···H17^xiii^	2.9453
C13···H8	3.319 (18)	H13···H25B^vi^	3.1443
C13···H9	3.2243	H16···O1^iii^	3.0762
C13···H10	2.78 (3)	H16···O4^iii^	2.6202
C14···H9	3.1969	H16···C26^viii^	3.2570
C14···H13	3.2053	H16···H1A^iii^	2.7913
C15···H1	2.4902	H16···H6A^i^	3.1682
C15···H3	2.6145	H16···H7B^i^	3.4437
C15···H22B	3.2173	H16···H26A^viii^	3.1956
C15···H25B	3.4305	H16···H26B^viii^	2.7439
C15···H25C	2.7652	H16···H26C^viii^	3.3177
C16···H17	3.2322	H17···C13^xiii^	3.2645
C17···H16	3.2321	H17···C17^xiii^	3.4602
C19···H1	3.2823	H17···H7A^vii^	3.1063
C19···H8	2.663 (19)	H17···H7B^vii^	3.4281
C19···H20A	3.2423	H17···H13^xiii^	2.9453
C19···H25A	3.4469	H17···H17^xiii^	3.3099
C19···H25C	2.7731	H17···H23A^xi^	3.5394
C20···H22A	3.2797	H17···H23C^xi^	2.9151
C20···H22B	2.7119	H17···H24C^xi^	3.4557
C20···H25A	3.3149	H17···H25B^vi^	3.0675
C20···H25B	2.6686	H20A···F1^iv^	2.8996
C20···H25C	2.6571	H20A···C24^ix^	3.3570
C20···H26A	3.3304	H20A···H6B^ix^	2.9153
C20···H26B	2.7584	H20A···H24A^ix^	3.2422
C20···H26C	2.6485	H20A···H24C^ix^	2.6614
C22···H1A	3.0318	H20B···O3^v^	2.7334
C22···H8	3.590 (18)	H20B···C2^v^	3.4491
C22···H20A	2.7190	H20B···H1^v^	3.2784
C22···H20B	3.2793	H20B···H3^v^	2.9758
C22···H25A	2.6944	H20B···H10^ix^	3.4585
C22···H25B	3.3258	H20B···H24A^ix^	3.1516
C22···H25C	2.6684	H20B···H24C^ix^	3.2052
C22···H26A	2.7448	H22A···H6A^ii^	3.1425
C22···H26B	2.6447	H22A···H6B^ii^	3.4254
C22···H26C	3.3257	H22A···H23B^ii^	3.2262
C23···H6A	2.5432	H22A···H23C^ii^	3.3559
C23···H6B	2.7395	H22A···H25A^iii^	2.7089
C23···H7A	2.7768	H22B···C6^ii^	3.3179
C23···H7B	2.5806	H22B···H6A^ii^	2.9929
C23···H24A	3.3302	H22B···H6B^ii^	2.7239
C23···H24B	2.6919	H23A···C7^xii^	3.3259
C23···H24C	2.6575	H23A···C18^xii^	3.5590
C24···H6A	3.3266	H23A···C23^xii^	3.2657
C24···H6B	2.6039	H23A···C24^xii^	3.5806
C24···H7A	2.6170	H23A···C25^iv^	3.5987
C24···H7B	3.3328	H23A···H7A^xii^	2.5240
C24···H23A	2.7323	H23A···H8^iv^	3.5752
C24···H23B	3.3254	H23A···H17^xiv^	3.5394
C24···H23C	2.6241	H23A···H23A^xii^	2.3987
C25···H8	3.411 (19)	H23A···H24B^xii^	2.8208
C25···H20A	3.3152	H23A···H25B^iv^	3.4980
C25···H20B	2.5823	H23A···H25C^iv^	3.0346
C25···H22A	2.6105	H23B···C5^iv^	3.3995
C25···H22B	3.3273	H23B···C8^iv^	3.3493
C25···H26A	2.5899	H23B···C9^iv^	3.1961
C25···H26B	3.3102	H23B···C25^iv^	3.2955
C25···H26C	2.6949	H23B···H8^iv^	2.7186
C26···H20A	2.6190	H23B···H9^iv^	2.9511
C26···H20B	2.7469	H23B···H22A^ii^	3.2262
C26···H22A	2.7291	H23B···H25A^iv^	3.1085
C26···H22B	2.6149	H23B···H25B^iv^	3.5664
C26···H25A	2.6442	H23B···H25C^iv^	2.7403
C26···H25B	2.6512	H23C···F1^xiv^	3.1345
C26···H25C	3.3080	H23C···C14^xiv^	3.5700
H1···H1A	3.5404	H23C···C17^xiv^	3.4005
H1···H3	1.9773	H23C···C25^iv^	3.2059
H1···H8	3.2400	H23C···H7A^xii^	3.3690
H1···H10	2.4367	H23C···H17^xiv^	2.9151
H1A···H8	2.9071	H23C···H22A^ii^	3.3559
H1A···H10	3.3743	H23C···H25A^iv^	2.9786
H1A···H22A	3.2484	H23C···H25B^iv^	3.0829
H1A···H22B	3.3076	H23C···H25C^iv^	3.0132
H3···H7A	3.2561	H24A···O2^vii^	2.7478
H3···H7B	3.3091	H24A···C1^vii^	3.4711
H6A···H7B	2.6557	H24A···C15^vii^	3.3645
H6A···H23A	3.4169	H24A···H1^vii^	2.5911
H6A···H23B	2.2912	H24A···H3^vii^	3.2648
H6A···H23C	2.8504	H24A···H20A^vi^	3.2422
H6A···H24A	3.5984	H24A···H20B^vi^	3.1516
H6A···H24C	3.5100	H24B···C1^vii^	3.4705
H6B···H23B	2.9667	H24B···C8^vii^	3.3632
H6B···H23C	2.6258	H24B···C10^vii^	3.3756
H6B···H24A	2.8206	H24B···H1^vii^	2.8692
H6B···H24B	3.5017	H24B···H8^vii^	3.3563
H6B···H24C	2.4427	H24B···H23A^xii^	2.8208
H7A···H23A	2.5778	H24B···H25C^vii^	3.3627
H7A···H23B	3.1479	H24C···F1^xiv^	2.9076
H7A···H24A	2.8632	H24C···O2^vi^	3.4056
H7A···H24B	2.4360	H24C···C20^vi^	3.3082
H7A···H24C	3.5057	H24C···H1^vii^	3.1795
H7B···H23A	2.7369	H24C···H17^xiv^	3.4557
H7B···H23B	2.4577	H24C···H20A^vi^	2.6614
H7B···H23C	3.4938	H24C···H20B^vi^	3.2052
H7B···H24B	3.5089	H25A···C22^iii^	3.5958
H8···H9	2.4215	H25A···C23^i^	3.4340
H8···H10	2.88 (4)	H25A···H22A^iii^	2.7089
H8···H13	3.5893	H25A···H23B^i^	3.1085
H8···H22A	3.5787	H25A···H23C^i^	2.9786
H8···H25A	3.5445	H25A···H25A^iii^	3.0256
H8···H25C	2.5769	H25A···H26A^iii^	2.8698
H9···H16	2.3086	H25B···O3^v^	3.2933
H10···H13	2.3260	H25B···C13^ix^	3.1280
H10···H24A	3.3387	H25B···C17^ix^	3.0778
H13···H17	2.3178	H25B···C23^i^	3.5689
H20A···H22B	2.6301	H25B···H7A^v^	3.0946
H20A···H25B	3.4985	H25B···H13^ix^	3.1443
H20A···H25C	3.5747	H25B···H17^ix^	3.0675
H20A···H26A	3.5269	H25B···H23A^i^	3.4980
H20A···H26B	2.5201	H25B···H23B^i^	3.5664
H20A···H26C	2.7765	H25B···H23C^i^	3.0829
H20B···H25A	3.4755	H25C···C23^i^	3.1025
H20B···H25B	2.4038	H25C···H7A^v^	3.2487
H20B···H25C	2.8185	H25C···H23A^i^	3.0346
H20B···H26A	3.5677	H25C···H23B^i^	2.7403
H20B···H26B	3.1360	H25C···H23C^i^	3.0132
H20B···H26C	2.5353	H25C···H24B^v^	3.3627
H22A···H25A	2.4463	H26A···C14^x^	3.4741
H22A···H25B	3.5021	H26A···C16^x^	3.2142
H22A···H25C	2.8446	H26A···H16^x^	3.1956
H22A···H26A	2.6175	H26A···H25A^iii^	2.8698
H22A···H26B	2.9612	H26A···H26A^iii^	2.9284
H22B···H25A	3.5263	H26B···F1^x^	3.2540
H22B···H25C	3.5821	H26B···O4^ix^	2.9496
H22B···H26A	2.9391	H26B···C14^x^	3.4324
H22B···H26B	2.3881	H26B···C16^x^	3.1474
H22B···H26C	3.4761	H26B···H6B^ix^	3.3576
H23A···H24B	2.5761	H26B···H16^x^	2.7439
H23A···H24C	3.0027	H26C···O4^ix^	2.8821
H23B···H24B	3.5851	H26C···C5^ix^	3.4528
H23B···H24C	3.5078	H26C···C8^ix^	3.5574
H23C···H24A	3.5058	H26C···C10^ix^	3.3555
H23C···H24B	2.8935	H26C···C13^ix^	3.5780
H23C···H24C	2.4234	H26C···H10^ix^	2.9353
H25A···H26A	2.3988	H26C···H16^x^	3.3177
H25A···H26B	3.4989		
			
C3—C1—C4	114.93 (13)	C11—C6—H6B	108.604
C3—C1—C10	112.04 (14)	C18—C6—H6A	108.596
C4—C1—C10	116.04 (18)	C18—C6—H6B	108.601
O3—C2—C3	122.72 (19)	H6A—C6—H6B	107.561
O3—C2—C7	115.6 (2)	C2—C7—H7A	108.609
C3—C2—C7	121.71 (15)	C2—C7—H7B	108.601
C1—C3—C2	119.27 (14)	C18—C7—H7A	108.608
C1—C3—C11	122.46 (19)	C18—C7—H7B	108.602
C2—C3—C11	118.26 (18)	H7A—C7—H7B	107.579
C1—C4—C15	119.16 (16)	C5—C8—H8	113.0 (15)
C1—C4—C19	123.12 (15)	C10—C8—H8	120.8 (15)
C15—C4—C19	117.58 (15)	C5—C9—H9	119.245
C8—C5—C9	119.27 (15)	C16—C9—H9	119.224
C8—C5—C13	123.0 (2)	C1—C10—H10	114.8 (16)
C9—C5—C13	117.69 (19)	C8—C10—H10	117.3 (15)
C11—C6—C18	114.65 (14)	C5—C13—H13	119.337
C2—C7—C18	114.6 (2)	C17—C13—H13	119.338
C5—C8—C10	126.20 (17)	C9—C16—H16	120.715
C5—C9—C16	121.53 (17)	C14—C16—H16	120.700
C1—C10—C8	127.65 (16)	C13—C17—H17	120.792
O4—C11—C3	122.67 (19)	C14—C17—H17	120.814
O4—C11—C6	115.41 (15)	C15—C20—H20A	108.602
C3—C11—C6	121.9 (2)	C15—C20—H20B	108.605
C5—C13—C17	121.3 (3)	C21—C20—H20A	108.614
F1—C14—C16	118.7 (3)	C21—C20—H20B	108.604
F1—C14—C17	118.79 (17)	H20A—C20—H20B	107.564
C16—C14—C17	122.5 (3)	C19—C22—H22A	108.521
O2—C15—C4	122.55 (17)	C19—C22—H22B	108.501
O2—C15—C20	115.74 (17)	C21—C22—H22A	108.517
C4—C15—C20	121.69 (19)	C21—C22—H22B	108.512
C9—C16—C14	118.6 (3)	H22A—C22—H22B	107.534
C13—C17—C14	118.39 (19)	C18—C23—H23A	109.468
C6—C18—C7	107.56 (17)	C18—C23—H23B	109.479
C6—C18—C23	108.35 (15)	C18—C23—H23C	109.468
C6—C18—C24	110.6 (2)	H23A—C23—H23B	109.472
C7—C18—C23	110.1 (2)	H23A—C23—H23C	109.462
C7—C18—C24	110.84 (13)	H23B—C23—H23C	109.478
C23—C18—C24	109.27 (18)	C18—C24—H24A	109.473
O1—C19—C4	124.01 (16)	C18—C24—H24B	109.467
O1—C19—C22	114.44 (17)	C18—C24—H24C	109.473
C4—C19—C22	121.55 (16)	H24A—C24—H24B	109.461
C15—C20—C21	114.63 (18)	H24A—C24—H24C	109.482
C20—C21—C22	107.8 (2)	H24B—C24—H24C	109.472
C20—C21—C25	108.55 (18)	C21—C25—H25A	109.474
C20—C21—C26	111.9 (2)	C21—C25—H25B	109.462
C22—C21—C25	109.5 (2)	C21—C25—H25C	109.465
C22—C21—C26	111.47 (16)	H25A—C25—H25B	109.488
C25—C21—C26	107.6 (3)	H25A—C25—H25C	109.479
C19—C22—C21	115.02 (16)	H25B—C25—H25C	109.459
C19—O1—H1A	109.456	C21—C26—H26A	109.470
C2—O3—H3	109.461	C21—C26—H26B	109.469
C3—C1—H1	104.006	C21—C26—H26C	109.472
C4—C1—H1	104.005	H26A—C26—H26B	109.472
C10—C1—H1	104.004	H26A—C26—H26C	109.471
C11—C6—H6A	108.608	H26B—C26—H26C	109.473
			
C3—C1—C4—C15	−93.59 (17)	C8—C5—C13—C17	178.82 (13)
C3—C1—C4—C19	82.0 (3)	C13—C5—C8—C10	26.9 (3)
C4—C1—C3—C2	91.11 (17)	C9—C5—C13—C17	0.4 (3)
C4—C1—C3—C11	−87.27 (19)	C13—C5—C9—C16	−0.3 (3)
C3—C1—C10—C8	−146.85 (13)	C11—C6—C18—C7	−47.8 (2)
C10—C1—C3—C2	−133.63 (14)	C11—C6—C18—C23	−166.88 (14)
C10—C1—C3—C11	48.00 (18)	C11—C6—C18—C24	73.35 (18)
C4—C1—C10—C8	−12.11 (19)	C18—C6—C11—O4	−160.81 (14)
C10—C1—C4—C15	132.97 (15)	C18—C6—C11—C3	20.9 (3)
C10—C1—C4—C19	−51.4 (2)	C2—C7—C18—C6	48.65 (17)
O3—C2—C3—C1	−8.3 (2)	C2—C7—C18—C23	166.54 (12)
O3—C2—C3—C11	170.12 (12)	C2—C7—C18—C24	−72.4 (2)
O3—C2—C7—C18	158.96 (13)	C5—C8—C10—C1	−173.56 (12)
C3—C2—C7—C18	−22.5 (2)	C5—C9—C16—C14	−0.0 (3)
C7—C2—C3—C1	173.21 (12)	C5—C13—C17—C14	−0.1 (3)
C7—C2—C3—C11	−8.3 (2)	F1—C14—C16—C9	−179.91 (14)
C1—C3—C11—O4	9.4 (3)	F1—C14—C17—C13	179.96 (14)
C1—C3—C11—C6	−172.44 (12)	C16—C14—C17—C13	−0.3 (3)
C2—C3—C11—O4	−169.00 (13)	C17—C14—C16—C9	0.3 (3)
C2—C3—C11—C6	9.2 (2)	O2—C15—C20—C21	−161.68 (15)
C1—C4—C15—O2	8.9 (3)	C4—C15—C20—C21	20.0 (3)
C1—C4—C15—C20	−172.97 (15)	O1—C19—C22—C21	159.85 (19)
C1—C4—C19—O1	−7.4 (4)	C4—C19—C22—C21	−21.1 (4)
C1—C4—C19—C22	173.69 (16)	C15—C20—C21—C22	−48.2 (2)
C15—C4—C19—O1	168.26 (18)	C15—C20—C21—C25	70.38 (18)
C15—C4—C19—C22	−10.7 (3)	C15—C20—C21—C26	−171.05 (14)
C19—C4—C15—O2	−166.96 (17)	C20—C21—C22—C19	48.9 (3)
C19—C4—C15—C20	11.2 (3)	C25—C21—C22—C19	−69.0 (3)
C8—C5—C9—C16	−178.84 (13)	C26—C21—C22—C19	172.0 (3)
C9—C5—C8—C10	−154.65 (15)		

Symmetry codes: (i) *x*, −*y*+1, *z*−1/2; (ii) −*x*+1, −*y*+1, −*z*+1; (iii) −*x*+1, *y*, −*z*+1/2; (iv) *x*, −*y*+1, *z*+1/2; (v) −*x*+1/2, *y*+1/2, −*z*+1/2; (vi) *x*, *y*−1, *z*; (vii) −*x*+1/2, *y*−1/2, −*z*+1/2; (viii) −*x*+1, *y*−1, −*z*+1/2; (ix) *x*, *y*+1, *z*; (x) −*x*+1, *y*+1, −*z*+1/2; (xi) *x*, −*y*, *z*−1/2; (xii) −*x*+1/2, −*y*+1/2, −*z*+1; (xiii) −*x*+1/2, −*y*+1/2, −*z*; (xiv) *x*, −*y*, *z*+1/2.

### Hydrogen-bond geometry (Å, º)

**Table:**

*D*—H···*A*	*D*—H	H···*A*	*D*···*A*	*D*—H···*A*
O1—H1*A*···O4	0.82	1.78	2.582 (2)	168
O3—H3···O2	0.82	1.85	2.652 (3)	168
C9—H9···O1^iii^	0.93	2.62	3.353 (3)	136
C16—H16···O4^iii^	0.93	2.62	3.499 (3)	158

Symmetry code: (iii) −*x*+1, *y*, −*z*+1/2.
